# Dissolution of EAF slag minerals in aqueous media: Effects of sonication on brownmillerite and gehlenite

**DOI:** 10.1016/j.ultsonch.2024.107065

**Published:** 2024-09-11

**Authors:** Recep Kurtulus, Mahtab Akbarzadeh Khoei, Elijah Damilola Adesanya, Juho Yliniemi

**Affiliations:** a Fiber and Particle Engineering Research Unit, University of Oulu, PO Box 4300 90014, Finland; b Department of Materials Science and Engineering, Faculty of Engineering, Afyon Kocatepe University, Turkey

**Keywords:** Electric arc furnace slag, Gehlenite, Brownmillerite, Dissolution, Sonication, Citrate

## Abstract

The accumulation of electric arc furnace slag (EAFS) in landfills has been causing severe environmental problems. This study examines the dissolution properties of EAFS minerals, including brownmillerite and gehlenite, essential for their possible use in resource recovery. An investigation was conducted to compare the effects of sonication and stirring on mineral dissolution while also assessing the usage of citrate as a complexing agent for gehlenite. Synthetic brownmillerite and gehlenite minerals were dissolved in aqueous solutions at room temperature using a 1:100 g/ml ratio. The dissolved elements were measured using Inductively Coupled Plasma Optical Emission Spectroscopy (ICP-OES), while zeta potential and X-ray Photoelectron Spectroscopy (XPS) were used to assess changes in surface chemistry. Brownmillerite had significant dissolution extents, with Al and Ca dissolving up to 16 % and 8 %, respectively, in contrast to gehlenite, which dissolved less than 2 % under similar conditions. Sonication significantly increased the dissolution of brownmillerite by up to 100 %, although its impact on gehlenite dissolution varied depending on the duration of time. Besides, adding citrate enhanced the leaching of Al and Ca from gehlenite by facilitating complexation. XPS data demonstrated differences in elemental ratios on brownmillerite and gehlenite surfaces affected by the method used and the presence of citrate. Lastly, the dissolution extents of Al and Ca from EAFS were up to 12 %, depending on time and mixing method, with a preference for sonication over stirring. In conclusion, this study showed that minerals in EAFS have distinct dissolution characteristics, and sonication and citrate can considerably enhance dissolution.

## 1 Introduction

Global demand for iron and steel has significantly driven industries to expand their production capacity [1], [2]. As steel production rates increase, so does the generation of slag—a byproduct of steel production. Electric arc furnace slags (EAFS), a primary type of slag from steel production, have recently risen scientific concern due to their growing accumulation in nature. Currently, EAFS generation reaches up to 100 million tons annually, requiring additional areas for storage [3], [4]. However, landfilling can cause severe ecological risks, such as leaching of heavy metals, alteration of soil chemistry, and contamination of underground water [5]. Therefore, finding ways to utilize EAFS in high-value applications, similar to blast furnace slag, which is another slag from steel manufacturing and used prevalently in cementitious applications, is crucial [6].

Typically, steel scraps and pig iron are the primary starting materials in the electric arc furnace process [7]. EAFS is an industrial byproduct after thermal processing and refining treatment. Due to the processing conditions, EAFS has a complex mineralogy comprising multiple crystalline compounds with a minimal amorphous phase [8], [9]. Major components of EAFS generally include calcium, aluminium, silicon, iron, and magnesium-bearing compounds such as gehlenite, brownmillerite, larnite, or wüstite [10], [11]. Until recently, many researchers have sought alternative applications for this complex-structured EAFS, such as road pavements and construction [12], [13], [14]. Despite valuable efforts, there remains a need for innovative approaches to exploit EAFS. In this regard, extracting elements such as aluminium, calcium, or silicon might be considered for future research because these elements may help develop possibilities in fields like carbon capture, cementitious binders, or catalysts [15], [16]. Consequently, a solid understanding of the dissolution properties of EAFS is essential to meet the requirements of these application areas.

Existing studies have focused on analyzing the dissolution properties of various inorganic byproducts (but less research on EAFS) in acidic, neutral, and alkaline environments to facilitate dissolution [17], [18]. Significant amounts of chemicals such as acetic acid (CH_3_COOH), ammonium chloride (NH_4_Cl), nitric acid (HNO_3_), or sodium hydroxide (NaOH) have been used in these investigations to extract elements [19], [20], [21]. However, high chemical consumption hinders the potential benefits of byproducts, making practical dissolution processes challenging. Thus, researchers have developed additional ways to minimize limitations and enhance dissolution characteristics.

In the dissolution process, researchers frequently use grinding and thermal activation as pretreatment methods, along with chemical methods, to enhance particle reactivity [22]. Recently, sonication has emerged as a viable strategy for increasing solute dissolution. Sonication involves stimulating ultrasonic waves from a source to form high-pressure and high-velocity bubbles in a liquid medium, depending on the device’s power (*e.g.*, ultrasound or megasound) [23], [24]. These bubbles can induce ultrasonic streaming, shock waves, or microjets, potentially causing damage to solid particles in the solution. This damage can increase particle fragmentation and surface defect formation, and thereby improving surface reactivity [25], [26]. Owing to these benefits, sonication can pave the way for improved elemental extraction, decreased chemical consumption, and green chemistry opportunities compared to conventional stirring.

Previous studies have explored the effects of sonication as an effective tool to improve dissolution properties. Arnold et al. [27] investigated the impact of cavitational stimulation using ultrasound and megasound on obsidian and calcite minerals, as well as fly ash, in aquatic environments. They found that both approaches increased mineral dissolution, with megasound significantly outperforming ultrasound compared to mechanical stirring. Another study by Dong et al. [28] examined the dissolution properties of blast furnace slag and fly ash using sonication and mechanical stirring techniques in water. Their research demonstrated that sonication could disrupt the atomic structure of the mineral, leading to increased structural breakdown. Additionally, Wei et al. [29] studied dolomite and quartz, Carletti et al. [30] explored limestone, Okumura [31] investigated lime, Said et al. [32] examined steel slags, and Rodriguez et al. [33] researched densified silica fume, all using sonication to improve reactivity and dissolution concentrations. Almost all studies indicated that the effectiveness of sonication in enhancing dissolution characteristics depends on factors such as materials mineralogy (crystalline or amorphous), structural integrity (bonding type such as Si–O), chemical environment (acidic, neutral, or alkaline), and sonication power (ultrasound or megasound). As a result, sonication shows great promise for improving element extraction and reducing chemical use.

Aside from studying the dissolution properties of various side streams and minerals, more research is needed on the influence of sonication, particularly for EAFS, owing to a research gap in the literature and the potential of EAFS for various applications. An in-depth investigation of the dissolution behavior of major mineral phases is also necessary to gain a deeper understanding of the overall dissolution of EAFS. Valuable insights have been achieved regarding the dissolution of minerals present in EAFS, such as mayenite, wüstite, and merwinite, through mechanical stirring under different chemical conditions [34], [35], [36]. Nevertheless, there remains a significant lack of understanding of other essential components, such as brownmillerite and gehlenite, when employing the sonication method. Considering the thermodynamic properties of brownmillerite and gehlenite based on the limited available data, it can be deduced that brownmillerite can be more reactive (or soluble in water) than gehlenite [37], [38]. With this in mind, studying the empirical dissolution characteristics of both synthetic slag minerals, therefore, can extend existing knowledge Since much more effort is needed over a wide range of experiments to determine solubility product constants (based on thermodynamic modeling) for both synthetic slags, the scope of the work was specifically canalized through experimental setups.

This research focused on dissolution studies of synthetically prepared brownmillerite and gehlenite minerals using mechanical stirring and sonication methods in an aqueous medium. Additionally, this study investigated the insertion of citrate as a dissolution-enhancing ligand that forms complexations on gehlenite while examining the dissolution characteristics of EAFS. The outcomes emphasized the potential of the sonication method to enhance the dissolution of EAFS under various criteria, thus enabling new opportunities for applications such as carbon capture or element extraction while mitigating ecological risks related to waste accumulation.

## 2 Materials & methods

### 2.1 Synthesis of minerals

The starting materials used were CaCO_3_ (VWR Chemicals, >99 %), Al_2_O_3_ (Merck,99.9 %), SiO_2_ (Sigma Aldrich, >99 %), and Fe_2_O_3_ (Alfa Aesar, 99.9 %). These materials were prepared into batch mixes based on the chemical composition of the minerals gehlenite and brownmillerite. To prepare the mixes, each material was weighed using an analytical scale (Ohaus, AX224, ±0.0001 g). The weighed materials were then mixed using planetary milling for 15 min to ensure thorough homogeneity. Subsequently, the mixes were individually transferred to platinum crucibles alloyed with rhodium and subjected to heat treatment in an Entech SF 6/17-S high-temperature furnace under normal atmospheric conditions. Table 1 provides the heating temperatures, rates, and dwelling periods required to synthesize the related minerals.

**Table 1:** Heat treatment conditions.

Mineral	Heating Temperature ( °C)	Heating Rate ( °C/min)	Dwelling Time (h)	Reference
Gehlenite	1400	5	72	[34]
Brownmillerite	1600	6	1	[39]

**Fig. 1:**
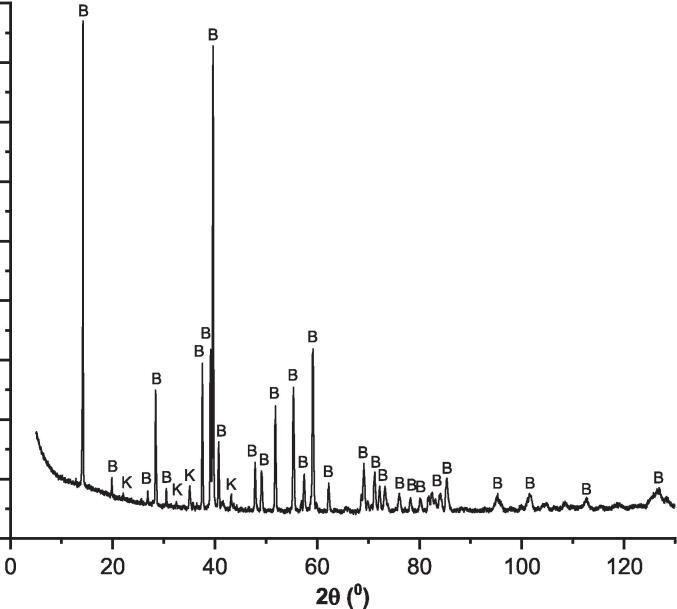
The XRD patterns of the synthesized brownmillerite (B: brownmillerite and K: krotite).

**Fig. 2:**
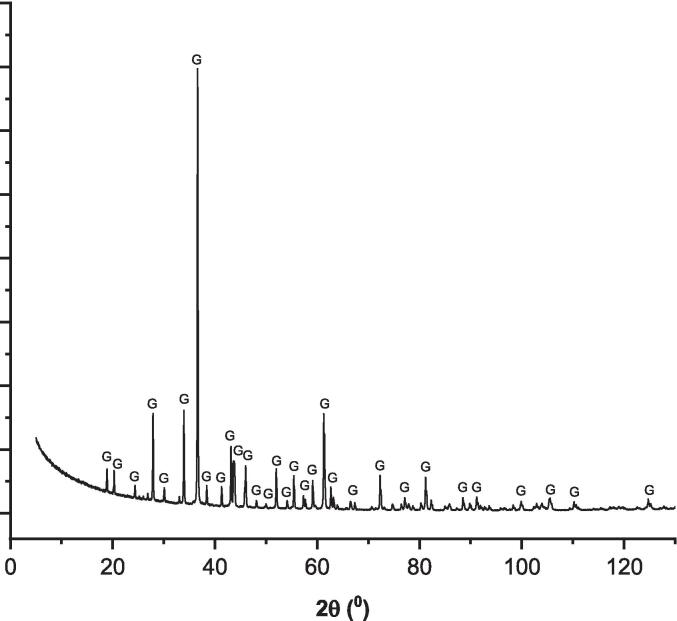
The XRD patterns of the synthesized gehlenite (G: gehlenite).

After subjecting the minerals to heat treatments, they were ground using an agate mortar and then screened through a sieve with a mesh size of 30 µm. Brunauer-Emmett-Teller (BET) method (via ASAP 2020, Micrometrics device) was then applied to the untreated powders to measure the specific surface area (SSA). The SSA for gehlenite and brownmillerite was 0.32 and 0.89 m^2^/g, respectively. The crystallographic phases and their respective quantities were determined using X-ray diffraction (XRD) with a 9 kW Rigaku Smartlab instrument, covering a range of 5°–130° and a step size of 0.02. The PDXL2 Software (with PDF-4 + 2023 database) was utilized for phase identifications. The resulting patterns are depicted in Fig. 1, Fig. 2, representing brownmillerite and gehlenite, respectively. Additionally, the phase quantification results for each synthetic mineral are shown in Table 2.

**Table 2:** The phase quantification of the synthesized minerals.

Phase	Gehlenite (%)	Brownmillerite (%)
Ca_2_FeAlO_5_ (pdf: 04-015-6868)	−	93.2
CaAl_2_O_4_ (pdf: 04-013-0779	−	6.8
Ca_2_Al[AlSiO_7_] (pdf: 04-010-5352)	100	−

### 2.2 Dissolution experiments

i Brownmillerite and gehlenite

A series of experiments facilitated the understanding of the dissolution behaviors of each mineral under Milli-Q-H_2_O conditions. Initially, the solid-to-liquid ratio was set at 1/100 g/mL. An analytical scale (Ohaus, AX224) was used to measure 1 g of each mineral with a tolerance of ±0.0001 g, while a graduated cylinder was employed to measure 100 mL of Milli-Q-H_2_O. Subsequently, two different techniques, mechanical stirring (MS) and sonication (S), were employed for mixing. It is important to note that both mixtures' velocities must be identical to accurately compare and evaluate the effectiveness of the approaches. To ensure this, Reynolds numbers (*Re*) were considered that the average circulation velocity (*v_C_*) led to normal flow (*Re*≈ 2000 to 4000), rather than laminar (*Re* ≤ 2000) or turbulent flow (*Re ≥* 4000), for both methods [40]. From these perspectives, the value of *Re* related to the MS method (*Re_MS_*) could be conveniently calculated using Equation (1).

(1) ReMS=νC1.L.ρn

where *ν_C1_*, *L*, *ρ,* and *n* are the average circulation velocity in MS (*m/s*), diameter of the stirrer bar (*m*), density of the liquid medium (*kg/m^3^*), and kinematic viscosity of the liquid medium (*kg/m.s*), respectively.

Contrary to the calculation of *Re_MS_*, determining *Re* for sonication (*Re_S_*) is somewhat more intricate. According to Refs. [24], [41], [42], estimating the value of *Re_S_* using Equation (2) is achievable.

(2) ReS=νC2.L.ρn

where *ν_C2_*, *L*, *ρ,* and *n* are the average circulation velocity in S (*m/s*), diameter of the horn tip (*m*), density of the liquid medium (*kg/m^3^*), and kinematic viscosity of the liquid medium (*kg/m.s*), respectively. One should apply Equation (3) to calculate the parameter *ν_C2_.*

(3) $$\nu_{C2}=\frac{5T+10Z}{\theta_{\min}}$$

where *T*, *Z*, and *ϴ_min_* represent the diameter of the beaker (*m*), height of the liquid in the beaker (*m*), and average mixing time (*s*), respectively.

These calculations indicated that *Re* (≈3880) can be comparable across both methods, suggesting that it is sufficiently similar to allow for accurate benchmarking.

**Fig. 3:**
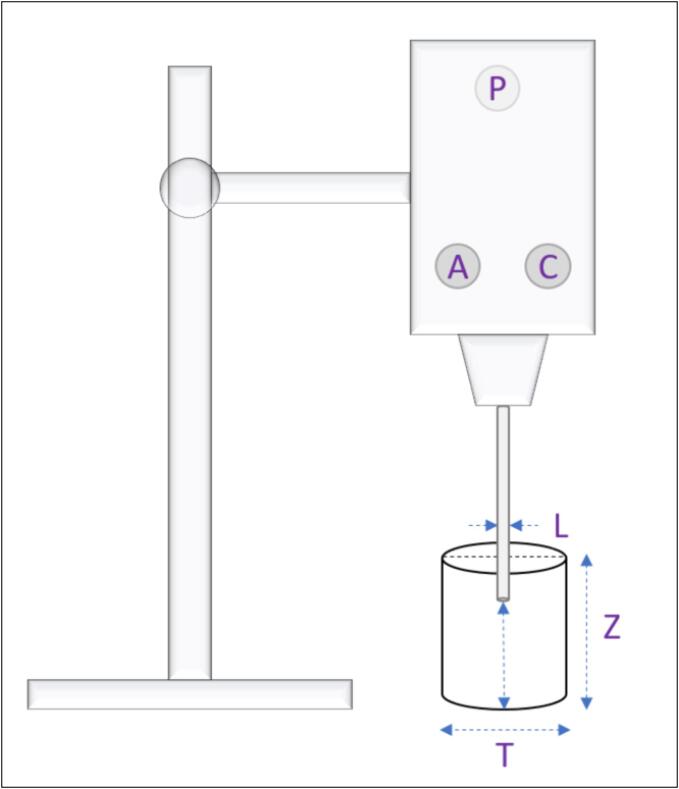
Visual representation of the sonication experiment (P, A, and C signify power, amplitude, and cycle parameters. Here, the power source is 400 W with 80 % efficiency.).

Within the scope of the MS experiments, the measured substances were blended in a beaker and agitated with a stirrer bar (L=0.015 m) at 330 rpm using the Velp Scientifica Arex Digital Pro apparatus for 30, 60, 90, and 120 min separately. The dissolution tests were performed at room temperature, 22 °C. For the S method, Fig. 3 shows the detailed experimental setup and the parameters that most impact the process. The weighed reagents were placed in another beaker (identical dimensions) and subjected to ultrasonic waves at 24 kHz using the Hielscher UP400S device for the same time allocations at room temperature, 22 °C. Sampling for ICP–OES, zeta potential, and pH analyses were conducted at regular time intervals for both techniques, as being described in the following section. The sample codes for MS and S experiments were determined as MS30 to MS120 and S30 to S120, respectively.

ii Citrate ligand

A 0.1 M trisodium citrate (hereafter citrate) solution was used instead of Milli-Q-H_2_O. The required quantity of citrate (99.9 % purity), purchased from Sigma Aldrich, was weighed, and mixed with 100 mL of Milli-Q-H_2_O to prepare the solution. Subsequently, the mixture was subjected to horizontal shaking for 24 h. The dissolution experiment for citrate use followed procedures employed in gehlenite study, including similar solid-to-liquid ratio and methodological approaches. The samples were labeled as G-MS30-C, G-MS60-C, G-S30-C, and G-S60-C to highlight the use of the ligand.

### 2.3 Characterisations

After completing the dissolution tests at each specified time, the related suspensions were vacuum-filtered using qualitative filter paper 413 (VWR, Ø- 75 mm) with a particle retention of 5–13 µm. Subsequently, a syringe equipped with a 0.22 µm syringe filter (VWR, Ø- 25 mm) was used to collect the leachate, which was then acidified using a 2 % HNO_3_ (Merck) solution. The acidified solutions were stored in a refrigerator at a temperature of 4 °C until analysis via ICP–OES, which evaluated for elemental concentrations of Al, Ca, Mg, Si, Fe, and Ti according to the requirements of EN ISO 11885. It is important to note that replicated tests demonstrated a ≤±10 % variation in the data obtained by ICP–OES. For this reason, error bars throughout the related plots represent ±10 %.

Based on elemental concentrations obtained from ICP–OES analysis, the extent of dissolution percentage (*ED*%) was calculated using Equation (4) [43], [44].

(4) $$ED\left(\%\right)=\frac{C_{i}.V}{m.x_{i}}.100$$

where *C_i_*, *V*, *m*, and *x_i_* are the concentration of element-*i* (mg/L) based on ICP–OES result, volume of the solution (L), mass of the EAFS in the solution, and mass fraction of element-*i* in the EAFS composition (based on XRF analysis), respectively. Here, it should be noted that *ED*% was only used to understand the changes in dissolution over time because precipitation and/or adsorption phenomena might occur along with the dissolution process, affecting the final dissolution extents.

After the dissolution tests and the collection of leachates, an additional syringe was used to reserve a 2 mL solution for zeta potential analysis. To counteract the ionic strength resulting from the dissolved species, a 0.1 mL solution of 0.1 M KCl was added to the aliquot solution as a background solution before conducting the zeta potential analysis. Subsequently, the solutions were transferred to disposable folded capillary cuvettes made of polycarbonate (DTS 1070). The zeta potential test was conducted using the Zetasizer Pro Blue instrument (ZSU5800 model, Malvern Pananalytical, UK) equipped with ZS Explorer software. The test was performed at 22 °C, with an equilibrium period of 60 s and a 30-s pause between each measurement. Three measurements were taken for each sample and median was calculated. It is important to note that the zeta potential test was conducted immediately following the completion of the dissolving experiment, which took approximately 15–30 min.

Before commencing pH measurement, buffer solutions pH 7 and 10 (VWR Chemicals) were used to calibrate the pH electrode. Then, the pH of the solutions following the dissolution tests were determined using an inoLab pH7110 equipment, with a margin of error of less than 0.1 pH units.

After vacuum filtration, the remaining solid particles on the filter paper were rinsed with distilled water and dried in the oven overnight. Then spectrum data between 4000 and 400 cm^−1^ were obtained by acquiring the powders using a Bruker Vertex v80 apparatus with a nitrogen-cooled MCT detector running in absorbance mode. The data were subsequently used to plot the trend between absorbance and wavenumber spectra.

The solid residues were stored in a desiccator until testing, *i.e.*, for X-ray photoelectron spectroscopy (XPS) analysis. The Thermo Fisher Scientific ESCALAB 250 Xi instrument, equipped with an Al-K_α_ X-ray source with an energy of 1486 eV, was used for spectral data acquisition. Scans were conducted using an energy of 150 electron volts (eV) with a step size of 1 eV. After the completion of the analysis, the collected data were assessed using Avantage Software. Additionally, the specific adventitious carbon (C 1 s, 284.8 eV) was calibrated for each spectrum before making the related assignments.

The synthetic slag minerals before and after dissolution experiments were analyzed using scanning electron microscopy (SEM) to observe the surface morphology. The particles were placed on a carbon plate and coated with carbon before analysis. A Zeiss Sigma FESEM device was used under 15 kV acceleration voltage.

## 3 Results

The results have been divided into two main sections, centered on brownmillerite (BM) and gehlenite (G) minerals. Each section includes details on dissolution characteristics, zeta potential and pH measurements, FTIR analysis, and XPS determination, offering a comprehensive characterisation of the features of both minerals.

### 3.1 Brownmillerite mineral

#### 3.1.1 Dissolution characteristics

**Fig. 4:**
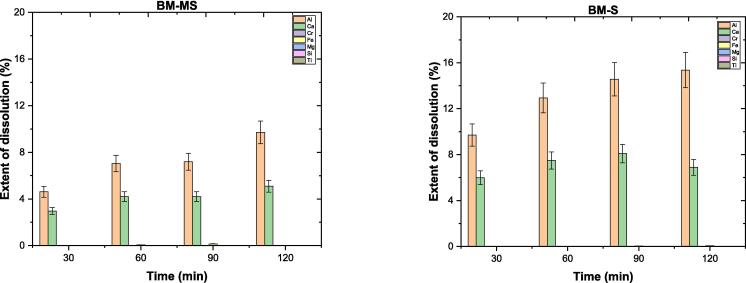
The extent of dissolution for elements in brownmillerite over time.

The assessment of BM dissolution characteristics involved evaluating dissolution concentrations (*DC*) and extent of dissolution (*ED*%) using ICP–OES data. The full data on *DC* for BM mineral is provided in the Supplementary Document, specifically in Figure S1. Additionally, Fig. 4 illustrates the *ED*% for elements in BM mineral over time, indicating the methods used. For Al, *ED*% increases from 4 to 10 % with MS and from 10 to 16 % with S over time. Meanwhile, for Ca, *ED*% transitions from 3 to 5 % with MS and from 5 to 7 % with S. These findings suggest that allocating more time combined with S can lead to increased elemental releases in the leachate.

#### 3.1.2 Zeta potential and pH measurements

**Fig. 5:**
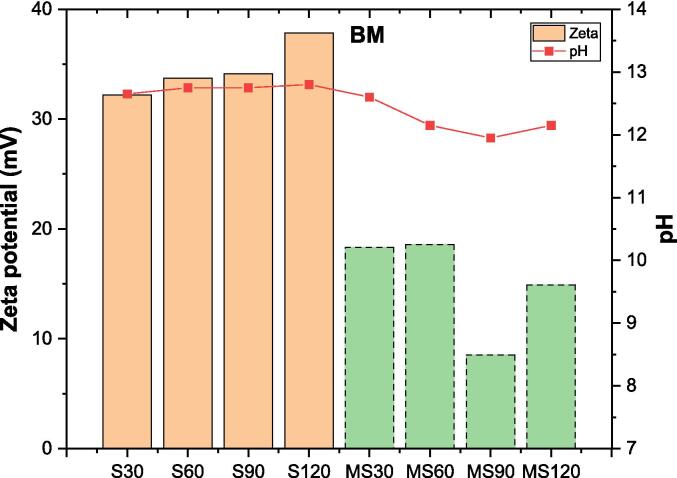
Zeta potential and pH of brownmillerite solutions over time.

Fig. 5 illustrates the zeta potentials of BM particles using MS and S methods, alongside their corresponding pH values. After S, the surface of BM particles becomes more positively charged compared to MS. The zeta potentials of samples subjected to S for 30–120 min fluctuate between +30 and +40 mV, while those treated with MS exhibit a range of +5 to +20 mV over the same duration. Zeta potentials show an increasing trend over time with S, whereas this trend is not observed with MS. The zeta potential remains relatively consistent for the first 60 min, then decrease abruptly at 90 min before increasing again towards the end. For both methods, pH levels vary slightly between 12.15 and 12.60 and between 12.65 and 12.80, respectively, depending on the time allocation. These results suggest that the solution treated by S is more positively charged compared to that generated by MS.

#### 3.1.3 FTIR analysis

**Fig. 6:**
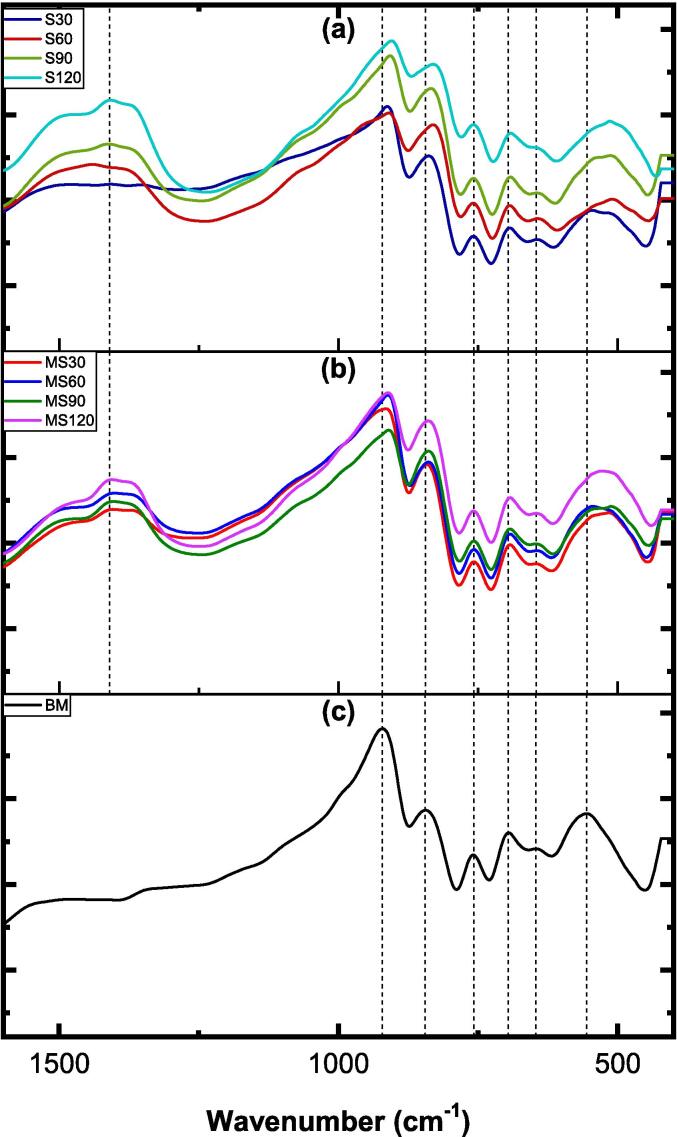
The FTIR spectra of untreated, mechanically stirred, and sonicated brownmillerite.

Fig. 6 displays the FTIR spectra, from 1600 to 400 cm^−1^, of the BM solid residue obtained using both methods, as well as its untreated form. For clarity, plots (a) and (b) correspond to S and MS experiments, while plot (c) is the untreated BM. The vertical dashed lines indicate the vibrational modes detected in the untreated BM.

The presence of O–H bonds in the untreated BM, potentially originating from the surrounding environment, is evidenced by a minor hump extending from 3600 to 3000 cm^−1^ (refer to Figure S2) [45]. Additionally, the identification of a narrow peak around 1600 cm^−1^ may suggest stretching and bending vibrations associated with H–O–H bonds [46]. In the fingerprint region (Fig. 6), a specific peak around 921 cm^−1^ may indicate the presence of Fe–O–Fe or O–Fe–O stretching units. Furthermore, the subsequent peak observed at 844 cm^−1^ could be linked to Al–O stretching vibrations [47], [48], while peaks at 757 and 696 cm^−1^ may be associated with Ca–O stretching units. The stretching vibrations of Fe–O bonds may also be represented by peaks at 646 and 555 cm^−1^ [49], [50].

Upon applying the proposed methods to dissolve BM, several minor and major peak shifts become evident. Plots (a) and (b) exhibit broader peak formations around 3600–3000 and 1640 cm^−1^ (Figure S2), indicating an increase in *T*–OH bonds (*T*: Al or Fe) on the surface owing to processing with water, *i.e.*, the surface becomes hydroxylated. There are some variations in the peak areas, for instance, in the S120 sample (sonication for 120 min), suggesting that *T*–OH bonds may be enhanced owing to the method used and duration. Another distinct peak formation, not present in BM mineral, is identified around 1425 cm^−1^ in almost all samples, associated with C–O vibrations resulting from carbonation of the surface by atmospheric CO_2_. The sharp peak formations observed in this study may be attributed to metal-carbonate vibrations, likely caused by the precipitation of calcium carbonates during the sample storage before analysis or after dissolution tests. Furthermore, notable peak shifts between 844 and 820 cm^−1^ and between 555 and 515 cm^−1^ may imply the presence of Al–OH and Fe–OH vibrational units, respectively, possibly resulting from the nucleophilic attack of hydroxyl ions on BM particles.

#### 3.1.4 XPS analysis

**Fig. 7:**
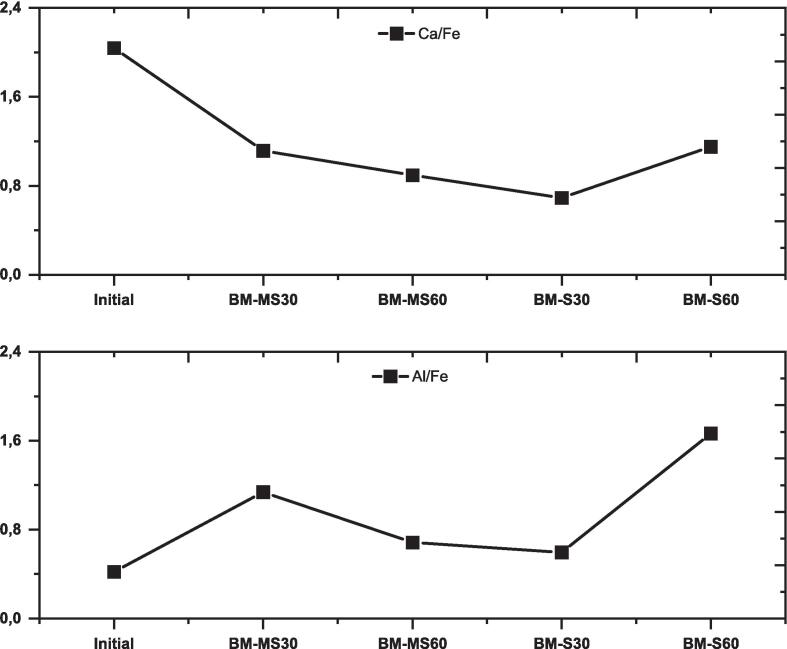
Changes in surface elemental ratios for brownmillerite mineral ('Initial' indicates the ratios based on XRF).

The surface composition of BM was analyzed using XPS technique for samples processed for 30 and 60 min. The surface elemental ratios, Al/Fe and Ca/Fe are depicted in Fig. 7. These ratios were chosen for consideration because Al and Ca are dissolved from the structure owing to processes such as proton–metal exchange reactions, while Fe remains in the structure (based on ICP-OES data). Thus, these ratios provide insight into the compositional changes occurring at the mineral surface. With the MS method (BM-MS30), the Al/Fe ratio initially increases after 30 min, followed by a decrease after the next 30 min, eventually approaching the initial ratio. In contrast, in plot (b), the Ca/Fe ratio in the MS method shows a decreasing trend over time, indicating a depletion of calcium from the surface. Moreover, both ratios on the BM surface decrease during the initial 30-min interval with S, followed by an upward trend after the 60-min mark. Although Al and Ca elements are initially released from the surface within the first 30 min via S, there may be surface accumulation (*e.g.*, adsorption or precipitation) of these elements after this period.

**Fig. 8:**
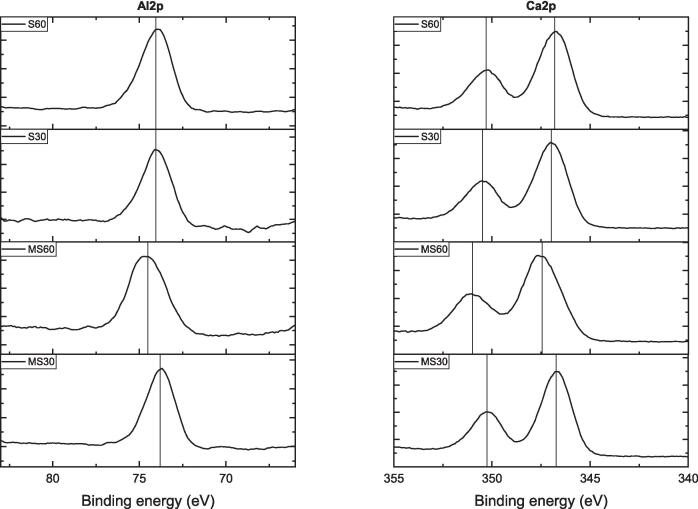
XPS spectra of Al2p and Ca2p for brownmillerite.

Fig. 8 shows the variations in peak binding energy (*PBE*) for Al2p and Ca2p, while Figure S3 (see Supplementary Document) displays Fe2p and O1s spectra over time using different methods. In Fig. 8, the Al2p peak shows a slight fluctuation around 74 eV, while Ca2p exhibits two peak positions at approximately 350 eV for Ca2p_3/2_ and 347 eV for Ca2p_1/2_. The Ca2p_3/2_ peak has a consistent *PBE* for all samples, except for MS60, where it shifts to 351 eV. After 60 min of MS, a slight *PBE* shift occurs for both Al and Ca elements.

#### 3.1.5 SEM analysis

**Fig. 9:**
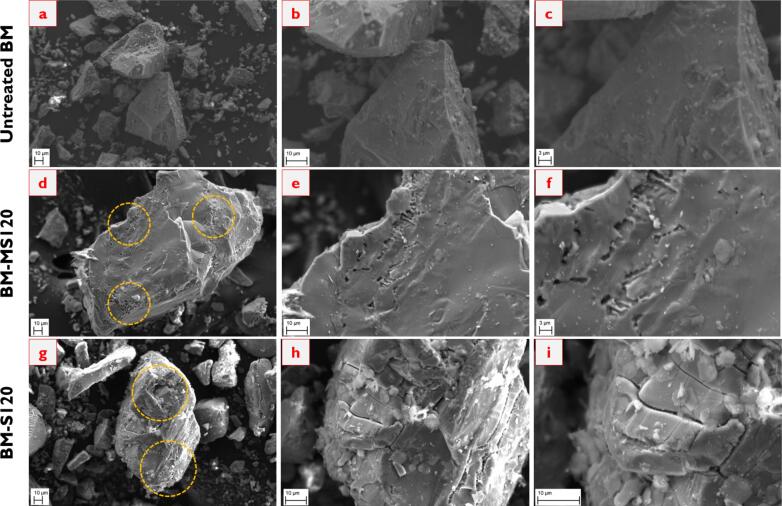
SEM images of the brownmillerite before and after dissolution (a – c: untreated BM; d – f: BM-MS120; g – i: BM-S120. Also, the magnifications are 1000X, 2500X, and 5000X from left to right.).

Fig. 9 demonstrates the surface morphology of the synthetic brownmillerite before and after dissolution experiments. The images extending from *a* to *c* represent the untreated BM with a smooth surface, sharp corners, and irregular shapes. Similar particle morphology was also observed by Ref. [51]. Following dissolution tests under mechanical stirring conditions, namely images *d* to *f*, the surface becomes dissolved as in the highlighted regions. Since the particles have sharp corners and irregular shapes, the dissolution process might initiate from these points. One may also observe pitting-like formations on the surface, possibly indicating the elemental releases. Besides that, the surface morphology after dissolution experiments under sonication conditions, the images from *g* to *i*, reveals much more surface deterioration. As given in the circled regions, the particle experiences micro-cracks due to applying sonication. Due to the impact of ultrasonic waves, the dissolution extent is enhanced, leading to more elemental releases originating from the damage created. From these findings, one can showcase the effect of the sonication technique on the particle surface, enabling more Al and Ca releases compared to the mechanical stirring technique.

### 3.2 Gehlenite mineral

#### 3.2.1 Dissolution characteristics

**Fig. 10:**
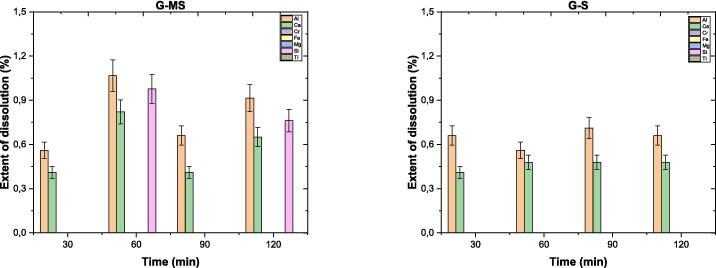
The extent of dissolution percentages of elements from gehlenite over time.

The dissolution process of gehlenite was investigated similarly to brownmillerite. The data on dissolution concentration (*DC*) of G is provided in the Supplementary Document, specifically in Figure S4. Fig. 10 illustrates the extent of dissolution (*ED*%) of elements in G over time. The *ED*% for Al ranges from 0.6 to 1.2 % and 0.6 % and 0.8 % with MS and S, respectively. There is a slight difference in *ED*% between the two methods at 30 and 90 min, with the S method showing a slight advantage over MS. However, MS demonstrates better *ED*% at 60 and 120 min. Additionally, comparable results have been observed for Ca in *ED*% across various time intervals using both methods, except for MS60. Overall, G exhibits limited dissolution extents (<2 %) under water conditions, regardless of the applied method.

#### 3.2.2 Zeta potential and pH measurements

**Fig. 11:**
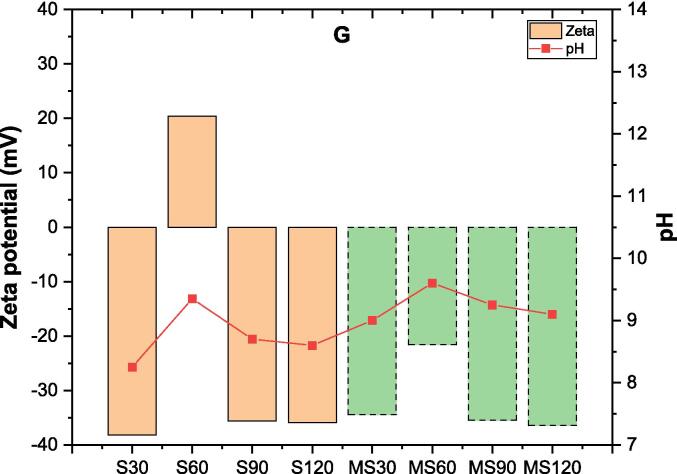
Zeta potential and pH of gehlenite over time.

Fig. 11 illustrates the changes in zeta potential and pH related to G following S and MS methods. The zeta potential remains consistently negative regardless of the method used, except for the S60 sample (sonication for 60 min). When subjected to S, G exhibits a zeta potential of approximately −35 mV, comparable to the results obtained with MS. However, S60 shows a zeta potential of +20 mV, whereas MS60 exhibits a less negative zeta potential of −21 mV compared to the other samples. This suggests that the surface charge may be influenced by protonation reactions after 60 min of dissolution with both methods. Moreover, the pH ranges between 8 and 9 for each sample, except for the S60 and MS60 samples, which range between 9 and 9.5.

#### 3.2.3 FTIR analysis

**Fig. 12:**
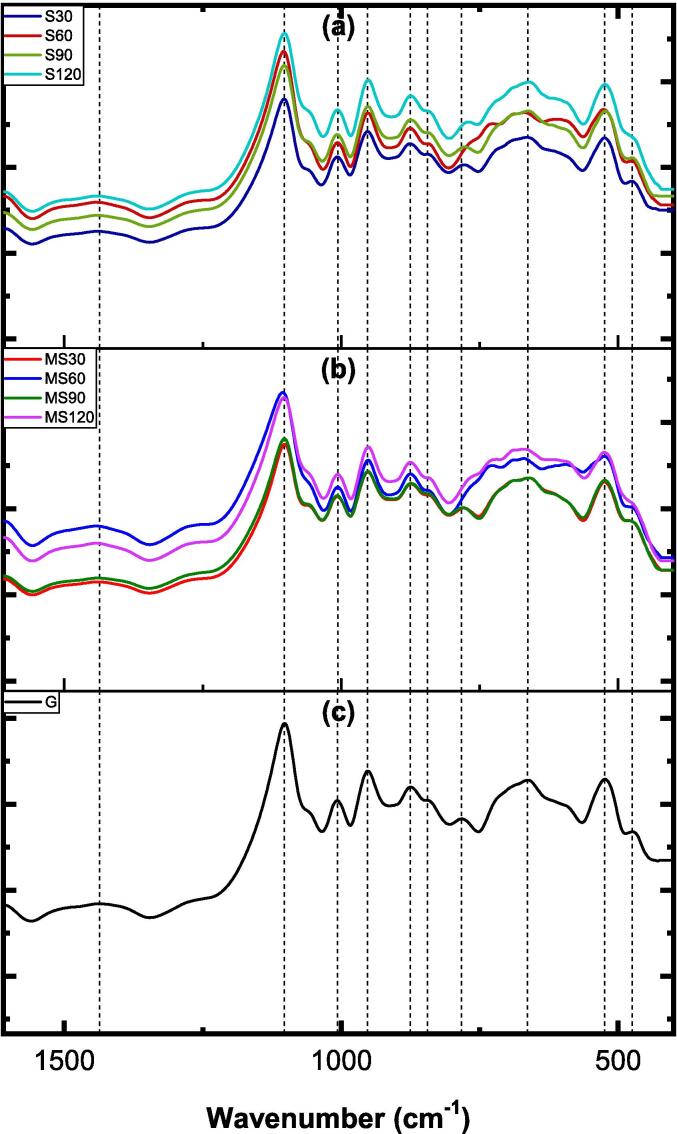
FTIR spectra of untreated and mechanically stirred and sonicated gehlenite.

Fig. 12 depicts the FTIR spectra of the G solid residue obtained using S and MS (plots a and b, respectively), alongside the untreated G material (plot c). The spectra show only minor changes, with no distinctive peak shapes or shifts compared to the untreated G material. There appears to be a potential correlation between O–H and H–O–H bonds, indicated by somewhat developed hump forms within the wavenumber range of 3600–3000 cm^−1^ and at 1600 cm^−1^, respectively (as revealed in Figure S5) [52]. Peaks observed at approximately 1440 cm^−1^ may be attributed to the stretching units of Si–O bonds [53], [54]. Further analysis suggests that peaks detected at approximately 1000 and 950 cm^−1^ correspond to molecular units of *T*–O–*T* (*T*: Al or Si) [55]. Moreover, the peak at 875 cm^−1^ may be associated with Al–O or Al–OH bonds [56]. Bending vibrations of *T*–O–*T* and *T*–O–Ca are likely observed at peak positions around 770 cm^−1^ [55], while peaks at approximately 525 cm^−1^ may be associated with *T*–O–*T* or *T*–O bending units [57]. These findings indicate that similar peak positions occur in G mineral regardless of the methods or time parameters employed.

#### 3.2.4 XPS analysis

**Fig. 13:**
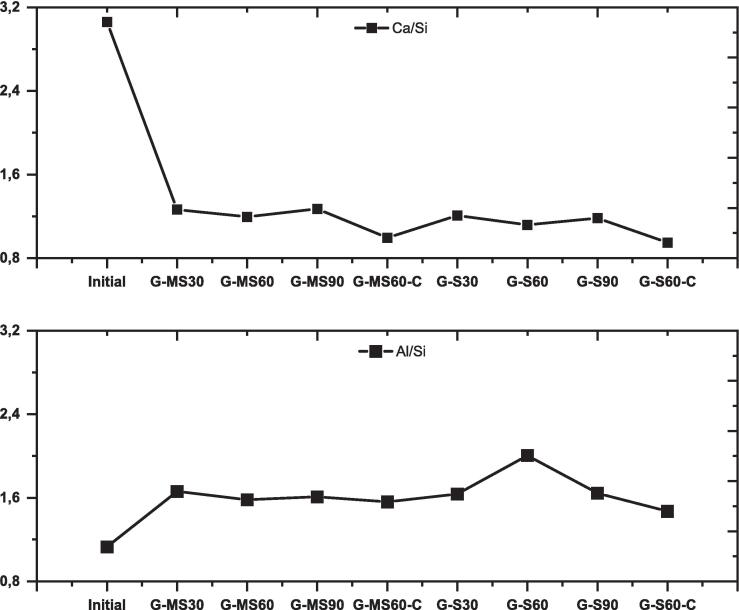
Variations in surface elemental ratios for gehlenite mineral with and without ligand use (‘Initial’ indicates the ratios based on XRF).

The surface chemistry of G mineral was analyzed using the XPS technique for 30, 60, and 90 min for each method. XPS spectra were evaluated based on the surface elemental ratio and peak binding energy. Fig. 13 shows the surface elemental ratio of Al/Si and Ca/Si, respectively. The Al/Si ratio increases from its initial level (*i.e.*, XRF ratio) and fluctuates within a narrow range when processed using the MS method. The Al/Si ratio for the S method follows a similar pattern to that of MS, with a significant deviation at 60 min, where the ratio progressively rises. This observation may suggest the occurrence of Al accumulation on the surface, potentially hindering the dissolution process via, for instance, a passivation layer [58]. When assessing the Ca/Si ratio using the MS method, it decreases up to 60 min and then returns to its initial value. For S, the ratio steadily decreases over time.

**Fig. 14:**
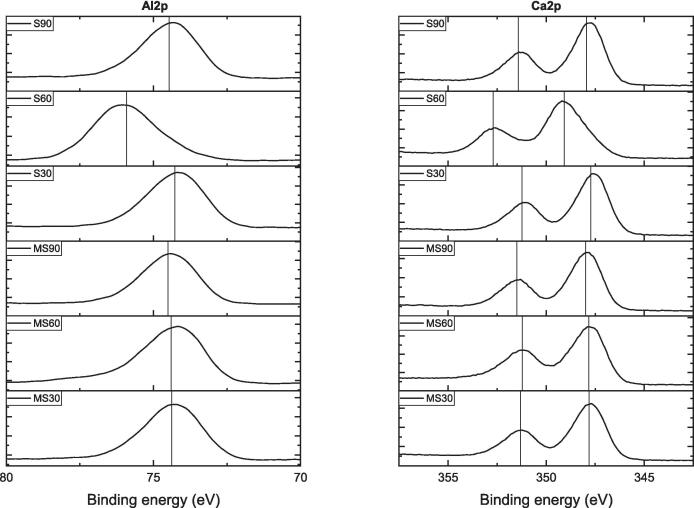
XPS spectra of gehlenite for Al2p and Ca2p peaks.

Fig. 14 displays XPS spectra illustrating the development of Al2p and Ca2p peaks. For Al2p, the MS series shows a *PBE* of 74.4 eV with minimal shift over time, while the S series is similar except for the S60 sample. The Al2p peak shifts to a higher *PBE* of 75.91 eV after 60 min of S. Additionally, the Ca2p peaks show two distinct peaks at 351 eV for Ca2p_3/2_ and 347 eV for Ca2p_1/2_. Similar to the Al2p peak shift with S60, the Ca2p peaks exhibit higher PBEs of 352.71 and 349.08 eV for Ca2p_3/2_ and Ca2p_1/2_, respectively. These results indicate that the S method, particularly at 60 min, increases the *PBE* of both elements.

Figure S6 shows the XPS spectra for the formation of Si2p and O1s during the experiments. Except for the S60 sample, which has a *PBE* of 103.88 eV, the MS and S series are identified within the Si2p peak formations at a *PBE* of 102 eV. This pattern holds for O1s, which appears at approximately 531 eV but shifts to 532.81 eV for the S60 sample. These findings suggest that using the S method for 60 min may cause the *PBE* of both elements in the G mineral to shift to slightly higher values.

#### 3.2.5 SEM analysis

**Fig. 15:**
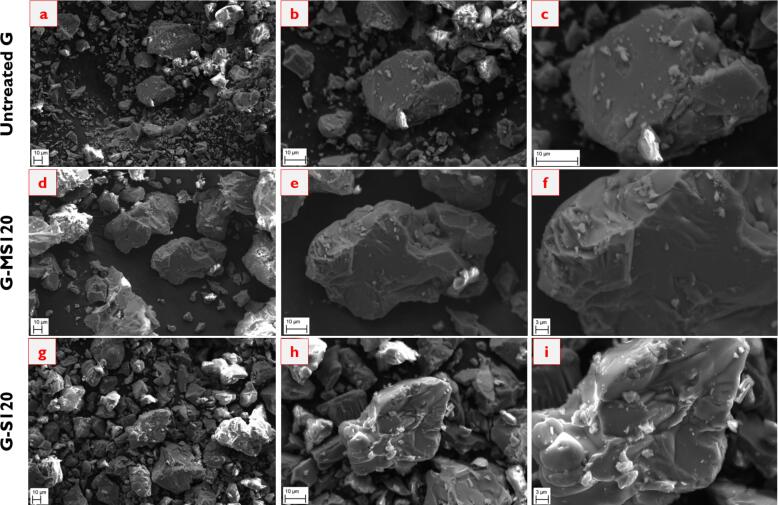
SEM images of the gehlenite before and after dissolution (a – c: untreated G; d – f: G-MS120; g – i: G-S120).

The surface morphology of the synthetic gehlenite before and after dissolution tests is presented in Fig. 15. It is essential to note that the images extending from *a* to *c*, *d* to *f*, and *g* to *i* indicate the untreated G, G-MS120, and G-S120 samples, respectively. The untreated G particles have a smooth surface and sharp corners, similar to the synthetic brownmillerite. The similar particle morphology can also be seen in the studies by Refs [59], [60]. When applying the mechanical stirring technique during the dissolution process, the particles do not show differences and resemble the untreated G. There are almost no micro-cracks and pitting-like defects on the surface due to the dissolution test. Likewise, one may not observe a reasonable change in the particle surfaces following the sonication technique. The particles are similar to the untreated G and the G-MS120 sample. It can only be said that the particle corners exhibit slight micro-cracks in the sonicated samples, but this may not be directly related to the effect of ultrasonic waves. Instead, it might have occurred because of particle grinding. The obtained surface morphology can be reasonable since the dissolution of gehlenite is very limited, irrespective of the applied technique. Nevertheless, it may be claimed that more time allowance may further initiate crack formation in the sonicated samples, leading to enhanced dissolution extents.

#### 3.2.6 Dissolution of gehlenite in the presence of citrate

**Fig. 16:**
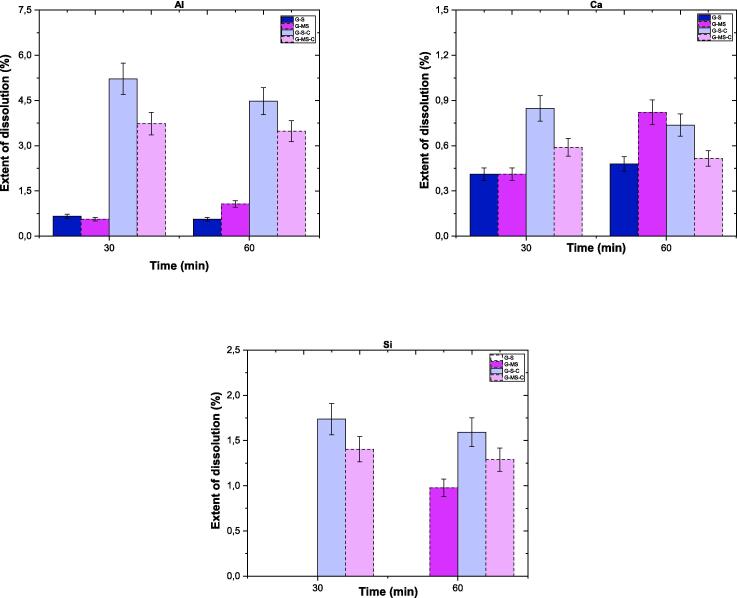
The extent of dissolution percentages of Al, Ca, and Si in gehlenite (with and without ligand) as a function of time (*e.g.*, G–S denotes sonicated G, while G–S–C represents sonicated G with the ligand citrate).

Because Al and Ca have been observed to accumulate on the surface of G (based on ICP–OES and XPS data), citrate was used as a complexing agent. Citrate is known to complex with Al, Ca, and other elements, preventing their precipitation on silicate surfaces and consequently increasing the dissolution of silicate materials [61], [62]. Based on the dissolution tests, Fig. 16 illustrates the *ED %* of Al, Ca, and Si over time for the samples treated for 30 and 60 min. The results reveal that the *ED %* of Al, Ca, and Si ranges between 0.5 % and 5.5 %, 0.3 % and 0.9 %, and 1 % to 2 %, respectively, depending on the specific method employed. Using the S method combined with citrate could significantly enhance the leaching of Al and Ca (*e.g.*, G-S-C series), resulting in several magnitude increments compared to the MS method having no citrate (*e.g.*, G-S series). Another noteworthy point is that Si is released into the solution in the presence of citrate when applying both methods. However, the combined use of sonication and citrate produced much more Si release. Furthermore, the zeta potential of S60 was + 20 mV, but in the presence of citrate, it became − 30 mV (S60-C in Figure S5), while the pH remained similar (∼8.5) in both samples.

The effect of citrate on surface chemistry based on XPS analysis is shown in Figure S8. The *PBE* of Al2p and Si2p, specifically for S60-C, exhibited a similar trend to the other samples, indicating a decrease compared to S60. Additionally, the samples treated with citrate solution had a Na1s peak (*PBE*: 1071 eV), indicating Na accumulation on the surface (because of using trisodium citrate), as depicted in Figure S9. Based on XPS analysis, Figure S10 presents the changes in the surface elemental ratios for G, both with and without the citrate. The surface shows the lowest Al/Si and Ca/Si ratios in the citrate-processed samples, which coincide with the highest dissolution percentages observed for Al and Ca. This demonstrates that citrate can complex Al and Ca in solution, preventing their surface accumulation (*e.g.*, adsorption or precipitation) and improving the dissolution of G.

## 4 Discussion

Brownmillerite (Ca_2_AlFe_2_O_5_) mineral has an orthorhombic structure consisting of alternating layers of FeO_6_ octahedral and FeO_4_ tetrahedral sites, with Al^3+^ tetrahedral sites and Ca^2+^ located between these layers [63]. Owing to its structural arrangement, Al^3+^ and Ca^2+^ are prone to leaching when reacting with water [64]. In the dissolution experiments, the initial pH of Milli-Q-H_2_O was approximately 7, indicating that the primary reaction pathway is proton–metal exchange reactions (1), (2), where two H^+^ release one Ca^2+^ and three H^+^ release one Al^3+^.

(1) Ca_2_AlFe_2_O_5_ + 4H_2_O → 2Ca^2+^ + H_4_AlFe_2_O_5_ + 4OH^−^

(2) H_4_AlFe_2_O_5_ + 3H_2_O → Al^3+^ + H_7_Fe_2_O_5_ + 3OH^−^

As these proton–metal exchange reactions consume protons, the pH increases from ∼7 to higher alkalinity levels (*i.e.*, >12). At high pH, the OH^−^ concentration becomes sufficient to dissolve Al through a hydroxyl mechanism, where OH^−^ ions attack the Al^3+^ tetrahedral sites, leading to the release of Al as Al(OH)_4_^−^.

According to the dissolution experiments, the concentrations of Al^3+^ and Ca^2+^ increase over time in brownmillerite. This means that longer reaction times lead to higher dissolution extents, with enhancements of ∼90 % and ∼50 % for Al^3+^ and Ca^2+^, respectively, from 0 to 120 min. Moreover, the extent of dissolution for both cations increases significantly (up to 100 % enhancement) when the sonication method is applied. The increasing dissolution extents for both cations are also reflected in the surface elemental ratios, specifically Ca/Fe and Al/Fe. A decrease in these ratios from their initial states indicates the release of elements into the solution. In contrast, an increase indicates the accumulation (*e.g.*, adsorption and/or precipitation) of Ca and Al on the surface, which may hinder dissolution by forming a passivating layer [21]. Our findings show a lower concentration of Al (as indicated by the Al/Fe ratio) on the surface via sonication compared to mechanical stirring after the first 30 min. Despite the efficiency of sonication within the first 30 min, both ratios increase after 60 min compared to the initial and 30-min data, indicating surface accumulations of Ca and Al. Overall, the dissolution of brownmillerite in Milli-Q-H_2_O conditions releases Al^3+^ and Ca^2+^ cations into the solution, with these concentrations increasing with longer reaction times and sonication. Although cation accumulation on the surface occurs after a certain period, sonication still results in higher elemental release into the solution than mechanical stirring. Hence, it can be said that sonic waves reduce the accumulation of Al on the surface. However, it is unclear whether sonic waves influence the Fe-rich brownmillerite surface to prevent Al accumulation or if Al adsorbs/precipitates on the surface, with sonic waves dispersing or breaking down these precipitates.

Gehlenite (Ca_2_Al[AlSiO_7_]), belonging to the mellite family, is categorized under the sorosilicate group characterized by tetrahedra within a silicate matrix [65], [66]. Its tetragonal crystal structure comprises aluminosilicate structural units (AlO_4_ and SiO_4_) with calcium occupancy in the interlayers, rendering it more resistant to chemical attacks compared to brownmillerite [67]. Indeed, the dissolution extents for both elements were below 2 % in gehlenite whereas was up to 16 % in brownmillerite. From a reaction mechanism standpoint, the dissolution of Al^3+^ and Ca^2+^ in gehlenite occurs through similar proton–metal exchange reactions as explained for brownmillerite (Equations (1), (2). During the dissolution tests, because protons are not consumed by Ca leaching to the same extent as in the case of brownmillerite, the pH did not increase above 9.5 (initial pH: ∼7), which consequently prevents Al and Si dissolution by OH^−^ attack, thereby keeping the overall dissolution low. This limited dissolution extent of gehlenite in Milli-Q-H_2_O with a comparable pH was also reported by Engström et al. [34], who concluded that Al and Si bound to the crystal structure need to dissolve first for the dissolution of Ca to continue.

The low *ED*% of elements from gehlenite limits meaningful comparisons between mechanical stirring and sonication methods. However, there was an interesting change in gehlenite dissolution and surface chemistry after 60 min. The G-MS60 sample exhibited the highest *ED*% of Al and Ca and the highest zeta potential (−18 mV, while for other samples, it was below −30 mV) despite no notable change in Ca/Si and Al/Si surface ratios. Conversely, for the G-S60 sample, the increased *ED*% did not appear, whereas pH and zeta potential values increased, in addition to a sharp increase in the Al/Si ratio. These outcomes directed the authors toward investigating the surface accumulation of cations on gehlenite particles because the elimination of the passivating layer may enhance dissolution. When citrate—an Al and Ca complexing ligand—was added to the system, the leaching of Al and Ca from gehlenite increased by several orders of magnitude, indicating that surface reactions significantly impact the dissolution of gehlenite [62], [68]. As a result, surface elemental ratios (Figure S8) revealed a decrease for both mechanically stirred and sonicated samples (*e.g.*, G-MS60-C and G-S60-C). In summary, time and different methods may not result in an increasing dissolution of gehlenite in Milli-Q-H_2_O due to its crystal structure. Still, citrate increases the dissolution of both elements (plus minor Si release), especially when combined with the sonication method, supporting the proposed efficiency of sonic waves.

## 5 EAFS dissolution tests

After elucidating the dissolution of synthetic minerals, the dissolution of EAFS (not synthetically prepared but supplied) was also investigated. Details regarding the preparation of EAFS powder, its corresponding chemical composition (Table S1), and mineralogical data (Figure S11) are provided in the Supplementary Document. In terms of mineralogical composition, EAFS consists of alite (Ca_3_SiO_5_), larnite (Ca_2_SiO_4_), magnesioferrite (MgFe_2_O_4_), mayenite (Ca_12_Al_14_O_33_), and wüstite (FeO), in addition to brownmillerite and gehlenite crystal phases. The dissolution experiment was conducted using a 1/100 g/mL ratio under similar conditions to the mineral tests.

**Fig. 17:**
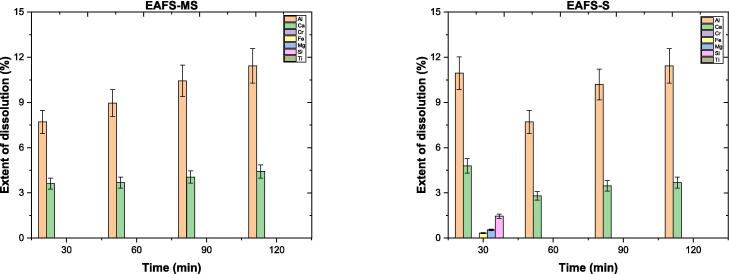
The extent of dissolution percentages of Al and Ca in EAFS over time.

The *ED %* for elements in EAFS are illustrated in Fig. 17. The *ED %* of Al and Ca exhibited reasonable variations over different time intervals, fluctuating around 7 % to 12 % and 3 % to 6 %, respectively, with both methods (slightly differing between MS and S methods, with S showing advantage). In addition to dissolving both elements via both methods, S method aided in dissolving other elements, such as Fe, Mg, and Si (<1.5 *ED %*), after the first 30-minute interval. Despite this, the subsequent time intervals did not result in the dissolution of these elements, possibly due to surface or solution accumulations. From the findings, while sonication significantly influences the dissolution of brownmillerite, other minerals in EAFS may not be as susceptible to it. Therefore, the overall impact of sonication on EAFS dissolution may remain low.

The zeta potential and pH of EAFS are shown in Figure S12. Regardless of the method used, the zeta potential of EAFS was slightly positive in all cases but much closer to neutral compared to BM and G minerals. This indicates that various minerals present in EAFS, such as larnite, wüstite, or mayenite, influence the zeta potential of EAFS particles [69], [70], [71]. The pH was approximately 12 with both methods, which is consistent with the dissolution extent results, considering that pH provides information about proton consumption and dissolution reactions, as discussed earlier. The solid residues were analyzed using the FTIR technique to identify structural vibrational modes. The spectra for all relevant samples are shown in Figure S13. In addition to the assigned peaks, as explained in the Supplementary Document, the peak positions in both sonicated and mechanically stirred samples are comparable to those identified in the untreated EAFS, regardless of the method employed. In conclusion, the dissolution of EAFS under Milli-Q-H_2_O conditions may be limited to the dissolution characteristics of individual minerals (*e.g.*, brownmillerite), and it may not exhibit significant changes with longer time allocations.

## 6 Conclusions

This study aimed to investigate the impact of sonication and mechanical stirring on the dissolution characteristics of two primary minerals, brownmillerite and gehlenite found in typical electric arc furnace slag. Batch dissolution experiments revealed that sonication increased the extent of dissolution of Al and Ca species from brownmillerite (with up to 100 % enhancement). However, its effect on gehlenite was less prominent or even decreased depending on the duration. Surface chemistry was assessed using XPS analysis, focusing on elemental ratios in brownmillerite (Al/Fe and Ca/Fe) and gehlenite (Al/Si and Ca/Si). Decreases in brownmillerite ratios after 30 min indicated higher dissolution, while an increased Al/Fe ratio after 60 min suggested surface accumulation of Al. Sonication reduced Al accumulation compared to mechanical stirring after 30 min. However, after 60 min, sonication increased the Al/Fe ratio, indicating higher accumulation, while mechanical stirring continued to decrease the ratios. The results indicated that sonication affected the dissolution and accumulation of Al and Ca on the brownmillerite surface, with a time-dependent impact. For gehlenite, a unique behavior of Al was observed during the experiments. After 60 min, mechanical stirring yielded the highest Al concentration in the solution, accompanied by a zeta potential shift from negative to positive, but no changes in the Al/Si ratio on the surface. Conversely, with sonication, Al dissolution remained lower, the zeta potential was negative, and the Al/Si ratio on the surface of gehlenite increased. The Ca/Si ratio of the gehlenite surface showed minimal variation throughout the experiments, regardless of the methods or time. Additional tests with trisodium citrate solution showed a sharp decrease in the Al/Si ratio of gehlenite, indicating that citrate complexed Al, hindering surface accumulation and consequently increasing dissolution by several orders of magnitude. Furthermore, the effect of mechanical stirring and sonication on the dissolution of electric arc furnace slag was similar for both methods, with slight variations depending on experimental time. These findings suggest that many minerals in electric arc furnace slag may not be susceptible to sonication-enhanced dissolution. In conclusion, brownmillerite and gehlenite exhibited distinct dissolution and surface reactions during experiments when treated with sonication. The final dissolution process is mainly influenced by several factors, including structural variations, time allocation, the presence of ligands, and the specific method employed.

## CRediT authorship contribution statement

**Recep Kurtulus:** Writing – review & editing, Writing – original draft, Visualization, Validation, Methodology, Investigation, Formal analysis, Conceptualization. **Mahtab Akbarzadeh Khoei:** Writing – review & editing, Investigation, Formal analysis. **Elijah Damilola Adesanya:** Writing – review & editing, Resources, Methodology. **Juho Yliniemi:** Writing – review & editing, Supervision, Resources, Funding acquisition, Conceptualization.

## Declaration of competing interest

The authors declare that they have no known competing financial interests or personal relationships that could have appeared to influence the work reported in this paper.

## Data Availability

The data will be available upon request.
