# Supplementary material for: Dissolution of EAF slag minerals in aqueous media: Effects of sonication on brownmillerite and gehlenite
Source: Ultrason Sonochem. 2024 Sep 11;110:107065. doi: 10.1016/j.ultsonch.2024.107065 (PMC11416688; doi:10.1016/j.ultsonch.2024.107065)
Supplement: Supplementary Data 1 [file mmc1.docx]

**S U P P L E M E N T A R Y D O C U M E N T**

**for**

**Dissolution of EAF Slag Minerals in Aqueous Media: Effects of Sonication on Brownmillerite and Gehlenite**

Recep Kurtulus ^1, 2, *^, Mahtab Akbarzadeh Khoei ^1^, Elijah Damilola Adesanya ^1^, Juho Yliniemi ^1^

^1^ Fiber and Particle Engineering Research Unit, University of Oulu, PO Box 4300, 90014, Finland

^2^ Department of Materials Science and Engineering, Faculty of Engineering, Afyon Kocatepe University, Türkiye

***Dissolution concentrations of brownmillerite***

**Figure S1** illustrates the concentration of dissolved elements over time related to brownmillerite mineral after completing mechanical stirring (MS) and sonication (S) methods. When comparing the dissolution concentration (*DC*) of the elements in both methods, Al, Ca, and Fe are identified as the dissolved species, although in varying amounts. During the 120 minutes period, the *DC*_Al_ in the MS method rises from 0.20 to 0.45 mmol/g, whereas *DC*_Ca_ varies within the range of 0.25 to 0.43 mmol/g. Fe slightly dissolves to 0.002 mmol/g after 60 minutes and rises to 0.005 mmol/g at 90 minutes. No *DC*_Fe_ is detected at 30 and 120 minutes. S method allows for achieving higher *DC* than MS, with at least a twofold increase for Al and Ca at all time intervals (e.g., 0.45 mmol/g for Al after 30 min). Unlike *DC*_Fe_ in MS, it produces reduced amounts at various time points, namely 0.001 mmol/g at 90 minutes and 0.002 mmol/g at 120 minutes.

**Figure S1.** Dissolution concentrations of elements related to brownmillerite.

***FTIR analysis of brownmillerite***

**Figure S2.** FTIR spectrum of brownmillerite solid residue after mechanical stirring and sonication methods.

***XPS analysis of brownmillerite***

**Figure S3** displays the XPS spectra of BM, showing the formation of Fe2p and O1s peaks. There are three dissimilar peak sites for Fe2p peaks: Fe2p_1/2_, Fe (III) Fe2p_3/2_ satellite, and Fe2p_3/2_ with *PBE* of 724, 717, and 710 eV, respectively. Although there are identical *PBE* formations of Fe2p_3/2_ and Fe2p_1/2_, subtle differences arise in the other formations due to distinct methods and time intervals. Using the S method, a minor change towards higher *PBE* is detected in the Fe (III) Fe2p_3/2_ satellite, from 717 to 718 eV. Additionally, the occurrence of O1s peak in BM arises at around 531 eV consistently across all samples, irrespective of the method used. Therefore, the MS and S methods may provide comparable peak formations, but the S method might yield a greater *PBE* for Fe2p.

**Figure S3.** XPS spectra of brownmillerite for Fe2p and O1s.

***Dissolution concentrations of gehlenite***

**Figure S4** exhibits Al, Ca, and Si elements as the dissolved species in gehlenite (G) mineral using mechanical stirring (MS) and sonication (S) methods. In the MS method, the *DC*_Al_ and *DC*_Ca_ species vary from 0.04 to 0.07 mmol/g and 0.03 to 0.06 mmol/g, respectively, during the experiment. Still, *DC*_Si_ is measured explicitly at 60 and 120 minutes, resulting in 0.04 and 0.03 mmol/g, respectively. Conversely, the S method produces comparatively poorer detection capability for Al and Ca elements and does not favor *DC*_Si_. *DC*_Al_ is primarily constant throughout time, but *DC*_Ca_ exhibits a modest rise.

**Figure S4.** Dissolution concentrations of elements related to gehlenite.

***FTIR analysis of gehlenite***

**Figure S5.** FTIR spectrum of gehlenite solid residue after mechanical stirring and sonication methods.

***XPS analysis of gehlenite***

**Figure S6.** XPS spectra of gehlenite for Si2p and O1s.

***Zeta potential and pH of gehlenite processed with and without citrate***

**Figure S7.** Zeta potential versus pH alterations in gehlenite (with and without ligand) as a function of time.

***XPS analysis of gehlenite processed with and without citrate***

**Figure S8.** XPS spectra of gehlenite (including citrate samples) for Al2p and Si2p.

**Figure S9.** XPS spectra of gehlenite for Na1s.

**Figure S10.** Variations in surface ratio of elements for gehlenite mineral with and without citrate ligand (‘C’) (‘Initial’ states the ratios based on XRF).

***Chemical composition and mineralogical data of EAFS***

The electric arc furnace slag (EAFS) originated from Finland. The grinding procedure (using planetary ball mill for 2 h) yielded particles with D10, D50, and D90 values of 0.63, 6.30, and 27.8 µm, respectively. The chemical composition of EAFS listed in **Table S1** was determined with X-ray fluorescence (XRF) technique with the Axios mAX device (Malvern PANalytical, UK). X-ray diffraction (XRD) technique using a 9 kW Rigaku Smartlab (10 to 130° with a 0.02° scan) device was, on the one hand, conducted to determine EAFS’s mineralogical composition, as shown in **Figure S11**. Further, PDXL2 software with PDF- 4+ 2023 database, was used for phase identifications. The matched main peaks are alite- A (pdf: 04-018-9701), brownmillerite- B (pdf: 04-010-5796), gehlenite- G (pdf: 04-014-7820), larnite- L (pdf: 04-007-9746), magnesioferrite- MF (pdf: 04-012-1070), Mayenite- M (pdf: 04-015-6170), and wüstite- W (pdf: 04-006-0816).

**Table S1**. Chemical composition of EAFS in wt.%.

| CaO | SiO_2_ | Al_2_O_3_ | Fe_2_O_3_ | K_2_O | MgO | P_2_O_5_ | TiO_2_ | SO_3_ | Cr_2_O_3_ |
| --- | --- | --- | --- | --- | --- | --- | --- | --- | --- |
| 38.00 | 14.78 | 7.60 | 27.27 | 0.03 | 8.00 | 1.19 | 0.654 | 0.60 | 0.068 |

**Figure S11**. The XRD patterns for the EAFS (A: alite, B: brownmillerite, G: gehlenite, L: larnite, MF: magnesioferrite, M: mayenite, and W: wüstite).

***Zeta potential and pH of EAFS***

**Figure S12.** Zeta potential versus pH alterations in EAFS as a function of time.

***Evaluation of the FTIR spectra for EAFS***

The spectra for all relevant samples are shown in **Figure S13**. For the sake of readability, it is essential to acknowledge that the plot- (a) illustrates the untreated EAFS, while the plots- (b)- and- (c) represent MS and S experiments, respectively. The vertical dashed lines also depict the vibrational modes identified in the untreated EAFS. When analyzing the FTIR spectra of the untreated EAFS, it becomes apparent that a broad peak ranging from 3600 to 3000 cm^-1^ may be ascribed to the presence of O-H bonds, potentially originating from the surrounding atmosphere [1]. Another peak has been observed at around 2500 cm^-1^, which could be attributed to atmospheric carbon dioxide (CO_2_^-^) [2]. In addition, detecting a peak at a wavenumber of 1633 cm^-1^ might indicate stretching and bending vibrations associated with H-O-H bonds [3]. Si-O stretching units, a common bond in silicate-containing systems, can be attributed to the strong peak at the wavenumber of 1454 cm^-1^ in the fingerprint region [4]. Furthermore, it is possible to credit the observed vibrational modes at 1074 and 968 cm^-1^ to the formations of Si-O-*T*, where *T* denotes either Si or Al [5], [6]. The identification of these units has often been reported in aluminosilicate structures. A peak at 858 cm^-1^ can be linked to the stretching units of Al-O bonds, whereas the peak at 727 cm^-1^ suggests the existence of bending units of Si-O-*T* bonds [7], [8].

**Figure S13.** FTIR spectrum of EAFS solid residue after mechanical stirring and sonication methods.

**REFERENCES**

[1] Y. X. Liu, F. Gu, H. Zhou, Q. Li, and S. Q. Shang, “Study on the performance and reaction mechanism of alkali-activated clay brick with steel slag and fly ash,” *Constr Build Mater*, vol. 411, Jan. 2024, doi: 10.1016/J.CONBUILDMAT.2023.134406.

[2] L. Kieush, J. Schenk, A. Koveria, and A. Hrubiak, “Insight into the Slag Foaming Behavior Utilizing Biocoke as an Alternative Carbon Source in Electric Arc Furnace-Based Steel Production,” *Journal of Sustainable Metallurgy*, 2024, doi: 10.1007/S40831-024-00783-9.

[3] M. Fang, Y. Yi, W. Ma, Y. Lin, J. Li, and W. Liu, “A study on the performance of alkali-activated materials prepared by thermochemical treatment of ladle furnace slag,” *Constr Build Mater*, vol. 411, p. 134560, Jan. 2024, doi: 10.1016/J.CONBUILDMAT.2023.134560.

[4] A. Elkhachine, N. Khachani, M. Saadi, and A. Diouri, “Mineralogy at early age of alkali activated mortar based on binary additions of limestone quarry dust and electric arc furnace slag,” *Mater Today Proc*, vol. 58, pp. 1566–1572, 2022, doi: 10.1016/j.matpr.2022.03.459.

[5] M. A. Gómez-Casero, S. Bueno-Rodríguez, E. Castro, and D. Eliche Quesada, “Alkaline activated cements obtained from ferrous and non-ferrous slags. Electric arc furnace slag, ladle furnace slag, copper slag and silico-manganese slag,” *Cem Concr Compos*, vol. 147, Mar. 2024, doi: 10.1016/J.CEMCONCOMP.2023.105427.

[6] A. Samadhiya, D. Bhunia, and S. Chakraborty, “Alkali-Activation Potential of Sandstone Wastes with Electric Arc Furnace Slag as Co-additive,” *Arab J Sci Eng*, 2023, doi: 10.1007/S13369-023-08514-0.

[7] S. K. Zakaria *et al.*, “Preliminary assessment of recycling Malaysia’s electric arc furnace (EAF) steel slag waste as one of raw materials for geopolymer ceramic product,” *Journal of Ceramic Processing Research*, vol. 22, no. 3, pp. 333–339, 2021, doi: 10.36410/jcpr.2021.22.3.333.

[8] A. Mishra, M. Lahoti, and E. H. Yang, “Mitigating environmental impact by development of ambient-cured EAF slag and fly ash blended geopolymer via mix design optimization,” *Environmental Science and Pollution Research*, 2023, doi: 10.1007/S11356-023-26884-8.
